# Neoadjuvant chemo-reirradiation followed by resection and intraoperative electron beam radiotherapy: outcomes of multimodality treatment for locally recurrent rectal cancer

**DOI:** 10.1186/s13014-025-02782-w

**Published:** 2025-12-23

**Authors:** F. E. C. Vande Kerckhove, F. Piqeur, E. Banken, N. C. Morsink, D. C. Rijkaart, J. S. Cnossen, R. H. N. Tijssen, C. C. A. Huibregtse Bimmel-Nagel, I. E. G. van Hellemond, J. Nederend, H. J. T. Rutten, J. G. Bloemen, J. W. A. Burger, A. E. Verrijssen, H. M. U. Peulen

**Affiliations:** 1 Department of Surgery, Catharina Hospital, PO Box 1350, Eindhoven, 5602 ZA The Netherlands; 2 GROW Research Institute for Oncology and Reproduction, Maastricht University, Maastricht, The Netherlands; 3 Department of Radiation Oncology, Catharina Hospital, Eindhoven, The Netherlands; 4 Department of Medical Oncology, Catharina Hospital, Eindhoven, The Netherlands; 5 Department of Radiology, Catharina Hospital, Eindhoven, The Netherlands

**Keywords:** Reirradiation, Locally recurrent rectal cancer, Acute toxicity, Late toxicity, IOERT, Local control, Survival, Multimodality treatment

## Abstract

**Background:**

Chemo-reirradiation has emerged as a feasible neoadjuvant therapy to improve resectability in locally recurrent rectal cancer (LRRC). However, its combination with surgery and intraoperative electron radiotherapy (IOERT) raises concerns regarding cumulative toxicity. This retrospective study aimed to evaluate acute and late toxicity profiles, local control and survival outcomes, following this multimodal approach in our institution.

**Methods:**

LRRC patients who underwent chemo-reirradiation and surgery with IOERT (median cumulative tumour dose of 113 Gy, α/β = 10 Gy) between September 2021 to December 2024 were retrospectively analysed. Acute and late treatment-related toxicities (CTCAE) were recorded in a prospectively maintained database. Secondary outcomes were overall survival (OS) and local re-recurrence-free survival (LRFS).

**Results:**

Among 40 patients, no grade 4 or 5 toxicities were observed. Acute cumulative treatment-related grade 3 toxicities occurred in 14/37 (38%) patients, predominantly consisting of erectile dysfunction (5/37, 14%), abscess formation (4/37, 11%) or peripheral neuropathy (2/37, 5%). Late grade 3 toxicities occurred in 13/30 (43%) patients, comprising mainly of erectile dysfunction (5/30, 17%), renal disorders (5/30, 17%) or peripheral neuropathy (2/30, 7%). After a median follow-up period of 21 months (IQR 12–32) after surgery, 2-year overall survival (OS) and local re-recurrence-free survival (LRFS) were 75.7% and 37.2%, respectively.

**Conclusion:**

In previously irradiated LRRC patients, a multimodality approach combining chemo-reirradiation and extensive surgery with IOERT demonstrated acceptable treatment-related toxicities and favourable oncological outcomes for this high-risk population. Further research with longer follow-up is warranted to identify risk factors associated with treatment-related toxicity.

**Supplementary Information:**

The online version contains supplementary material available at 10.1186/s13014-025-02782-w.

## Background

Introduction of total mesorectal excision and neoadjuvant (chemo)radiotherapy for intermediate-risk and locally advanced rectal cancer has significantly reduced recurrence rates to 6–10% [1–7]. Despite advancements in multimodal treatment, locally recurrent rectal cancer (LRRC) remains a complex disease, associated with substantial treatment-related morbidity, poor survival and diminished quality of life [8–10].

Achieving a radical (R0) resection during curative salvage therapy for LRRC is the most important prognostic factor for both overall survival and locoregional control [11–14]. However, this is often complicated by the infiltrative nature, altered anatomy and fibrosis from previous surgery and radiotherapy. Consequently, the Dutch National Guidelines recommend neoadjuvant chemoradiotherapy to facilitate tumour downstaging and enhance the likelihood of R0 resection [15].

In previously irradiated patients, chemo-reirradiation that has geometrical overlap with the irradiated volume of previous courses [16] presents a challenge in balancing effective locoregional control with acceptable treatment-related toxicities [15, 17, 18]. Although prospective data are lacking, retrospective studies suggest that in surgically treated patients, chemo-reirradiation can yield favourable tumour response with reasonable rates of local control, tolerability and toxicity [13, 19–25]. Nonetheless, the total dose deliverable with chemo-reirradiation remains limited by the cumulative dose tolerance of surrounding normal tissue. To address this, intraoperative radiotherapy with electrons (IOERT) enables targeted high radiation dose delivery, while positioning organs at risk (OAR) out-of-field to minimize toxicity [12, 26–28]. This approach aims to treat potentially involved resection margins intraoperatively and reduce local re-recurrence rates. Since 1994, our tertiary care referral centre has implemented IOERT for patients with LRRC [29]. From 2021 onwards, a revised dose-implementation technique has been adopted as routine practice to deliver a higher surface dose comparable to intraoperative brachytherapy (IOBT) [30, 31], alongside the initiation of a prospectively maintained toxicity database for continuous quality monitoring.

Large retrospective series from our centre on chemo-reirradiation for LRRC have demonstrated feasibility and acceptable toxicity [13, 23]. However, concerns persist regarding cumulative treatment-related toxicity in LRRC patients undergoing multimodality treatment with chemo-reirradiation, dose-adapted IOERT and surgical resection. This is in part due to limited systematic toxicity documentation. Given the established safety of chemo-reirradiation alone [13, 23], our prospectively maintained toxicity database focuses specifically on the cumulative treatment-related toxicity following this multimodality approach, which represents a key concern in current clinical practice. Using these data, the present study aims to evaluate acute and late treatment-related toxicity and oncological outcomes for LRRC patients managed with multimodality treatment in our tertiary referral centre.

## Methods

### Patient selection

All patients who underwent surgery with IOERT and were treated with neoadjuvant chemo-reirradiation for LRRC because of previous radiotherapy for the primary tumour at Catharina Hospital Eindhoven, a national tertiary referral centre for LRRC, from September 1, 2021 to December 31, 2024 were selected. The recent time period was chosen to ensure systematic treatment-related toxicity registration. All patients were discussed within an expert multidisciplinary team (MDT) including a radiation oncologist, surgical oncologist, medical oncologist, gastroenterologist, radiologist, and a pathologist. LRRC was defined as recurrence or development of new sites of tumor in the pelvis after previous total or partial mesorectal resection. Neoadjuvant treatment consisted of chemo-reirradiation with concomitant capecitabine. Selected patients received induction chemotherapy prior to chemo-reirradiation, generally consisted of either 3–4 courses CAPOX or 4–6 courses of FOLFOX or FOLFIRI. Patients with a re-recurrence were excluded. Baseline characteristics, neoadjuvant therapy, surgery, IOERT, post-operative complications, acute and late toxicities, and oncological outcomes were recorded. Follow-up was completed until June 24th, 2025. The study was approved by the Dutch Medical Research Ethics Committees United Nieuwegein (No.:AW25.011/W22.236).

### Chemo-reirradiation planning and delivery

For the primary tumour, patients received either short-course radiotherapy (5 × 5 Gy) or full course chemoradiotherapy (25 × 2 Gy with concurrent capecitabine) according to our national treatment and target delineation guideline [32]. For recurrent tumours, delineations and treatment plans were reviewed by a dedicated radiation oncologist with expertise in reirradiation for LRRC. A planning CT with oral contrast and full bladder protocol was acquired in supine position. For delineation, image registration of the planning CT with diagnostic MRI and/or PET-CT was strongly recommended. Delineation of gross tumour volume included all macroscopically visible tumour in primary staging, visible involved areas of surrounding organs and complete fibrotic areas. A clinical target volume (CTV) margin of 10 mm was employed with no adjustments towards other organs. Planning target volume (PTV) margins of 5–10 mm were used. Chemo-reirradiation consisted of a total dose of 30 Gy in 15 daily fractions of 2.0 Gy per fraction, using a volumetric modulated arc therapy or intensity-modulated radiation technique, with concurrent oral capecitabine (825 mg/m^2^) twice daily on the days of radiation therapy. Daily online cone beam CT was performed. There were no mandated OAR dose-constraints, with the priority being target coverage. The order of priority for optimisation in decreasing priority was CTV, PTV, small bowel, bladder and other OAR. Local restaging, consisting of a pelvic MRI and thoracoabdominal CT-scan, was performed between 4 and 6 weeks after the last day of chemo-reirradiation.

### Surgery

Type and extent of surgery was determined within the MDT. Surgery was categorised as: abdominoperineal resection (APR), low-anterior resection (LAR), total exenteration (TE, including resection of the rectum, bladder, prostate and vesicles in male patients, or ovaries, vagina and uterus in female patients) and tumour resection not otherwise specified (n.o.s., i.e., tumour resection without formal bowel resection). Additional resections performed during surgery were documented.

### Intra-operative electron radiotherapy

Preliminary IOERT indication was assessed within the MDT if resection margins were deemed at-risk, based on mesorectal fascia involvement, extramural vascular invasion or tumour adherence to adjacent structures on diagnostic MRI. Perioperatively, IOERT indication was re-evaluated by the radiation oncologist and surgical oncologist. A dose adaptation technique was developed and implemented as the standard of care at our centre from September, 2021, onwards [30, 31]. Without the bolus the dose at the tissue surface is approximately the same as the dose at prescription depth, being 10 Gy at 9 mm when using 6 MeV. By placing a bolus made of tissue-equivalent polymethyl methacrylate to the IOERT applicator exit, the maximum dose was transported to the tissue surface [31]. By additionally increasing the number of delivered monitor units the dose at tissue surface is increased to approximately 16 Gy while the dose at prescription depth, e.g., 9 mm for 6 MeV, remains 10 Gy. Thus, the depth dose profile within the patient was altered to approximate that of IOBT [31]. IOERT was administered using a Mobetron 2000 self-shielding mobile linear accelerator (IntraOp Medical, Sunnyvale California, USA) with applicator diameter ranging between 5 and 7 cm and a bevel angle of 45 degrees.

Total treatment dose was calculated as the sum of primary tumour radiation and current LRRC reirradiation (chemo-reirradiation and IOERT). The cumulative delivered dose was calculated by a radiation therapy technologist using the equivalent uniform dose in 2-Gy fraction (EQD2Gy), according to the following formula: EQD2Gy = $\:D\:\times\:\:\left\{d+\:\left(\alpha\:/\beta\:\right)\right\}/\left\{2Gy+(\alpha\:/\beta\:)\right\}$. $\:D$ refers to the total treatment course dose, $\:d$ to the fraction dose, and $\:\alpha\:/\beta\:$ equals 10 Gy.

### Follow-up and toxicity

All patients underwent follow-up according to the Dutch national guidelines [33]. From September 1, 2021, evaluation of treatment-related toxicities following multimodality treatment was prospectively registered by a radiation oncologist at 3 and 12 months post-operatively via teleconsultations and classified using the common terminology criteria for adverse events (CTCAE), version 5.0 [34]. If incomplete, patient records were retrospectively reviewed to assess evidence of toxicities. Toxicities related to chemo-reirradiation alone were also assessed retrospectively from medical records and correspondence letters. Post-operative complications and readmissions within 30 days were registered retrospectively using Clavien-Dindo classification [35].

### Outcomes

The primary study outcomes were acute (3 months) and late (12 months) treatment-related toxicities. Secondary outcomes were local re-recurrence-free survival (LRFS) and overall survival (OS). OS was calculated from the date of surgery until the date of death or censored at the last follow-up. LRFS was calculated from the date of surgery until the date on which local re-recurrence was detected by imaging or histology, or censored at the last follow-up, or death.

### Statistical analyses

Continuous data were calculated and expressed as median with interquartile ranges (IQR) or mean (± standard deviation), as appropriate. Categorical data were reported as count and percentages. OS and LRFS data were analysed using the Kaplan-Meier survival method. Due to the limitation in sample size and large number of substantial predictors, no analyses were performed to identify prognostic significant factors. All statistical analyses were performed using SPSS Statistics 29.0 (IBM Corp. Released 2022, Armonk, NY) and GraphPad Prism version 10.4 (GraphPad Software Inc., San Diego, CA).

## Results

### Patient characteristics

Between September 1, 2021 and December 31, 2024, 40 LRRC patients underwent surgery with IOERT after neoadjuvant chemo-reirradiation. Study cohort characteristics are presented in Table 1. Median follow-up was 16 months (IQR 10–30 months). The median interval between prior radiotherapy and chemo-reirradiation was 28 months (IQR 18–55). Reirradiation primarily consisted of 15 × 2 Gy (95%). One patient received 15 × 2 Gy and a targeted boost (15 × 3.1 Gy, EQD2Gy 50.8 Gy, α/β = 10 Gy) on two unresectable FDG-avid lymph nodes at the L5 level. Another patient received a modified compensatory regimen of 10 × 3 Gy, as concomitant capecitabine was declined by the patient.

**Table 1:** Tumour and neoadjuvant treatment characteristics

		*n* = 40	%
Gender	Male Female	30 10	75 25
***Primary tumour characteristics***			
Primary tumour	Rectum Rectosigmoid	39 1	98 2
Induction chemotherapy	No Yes	31 9	78 22
Induction chemotherapy, type	CAPOX FOLFOXIRI	8 1	89 11
Neoadjuvant RT	25 × 2 Gy 5 × 5 Gy Other*	24 14 2	60 35 5
Initial surgery	LAR APR Posterior exenteration	25 14 1	63 35 2
ypT stage	T0-T2 T3 T4	14 23 3	35 58 8
ypN stage	N0 N+	25 15	63 38
***Recurrent tumour characteristics***			
Age at diagnosis recurrence	Mean (SD)	64 (8.6)	
WHO score at diagnosis LRRC	0 1 2	31 8 1	78 20 3
Comorbidities	None 1 comorbidity 2 comorbidities ≥ 3 comorbidities	6 18 14 2	15 45 35 5
Primary-recurrence interval (months)	Median (IQR)	20 (10–48)	
Complaints at diagnosis LRRC	No Yes	22 18	55 45
Complaints at diagnosis LRRC, type	Pain Changed defecation pattern Wound complications, fistula or abscess Blood loss	6 7 3 2	33 39 17 11
Primary compartment LRRC	Central Posterior Lateral Anterior	21 9 8 2	53 23 20 5
Multifocal recurrence	No Yes	30 10	75 25
Induction chemotherapy	No Yes	27 13	68 33
Induction chemotherapy, type	CAPOX FOLFIRI	12 1	92 8
Interval between RT and reRT (months)	Median (IQR)	28 (18–55)	
Neoadjuvant reRT	15 × 2 Gy 10 × 3 Gy 15 × 2 Gy + boost	38 1 1	95 3 3
Neoadjuvant reRT, volumes**	Median GTV cm^3^ (IQR) Median CTV cm^3^ (IQR) Median PTV cm^3^ (IQR)	50 (18–120) 253 (119–424) 545 (338–857)	
Consolidation chemotherapy	No Yes (FOLFOX)	39 1	98 2
Metastases at diagnosis	No Yes	38 2	95 5
Interval reRT to IOERT (weeks)	Median (IQR)	13 (11–14)	

Due to rounding, not all percentages added up to 100%. Abbreviations: LAR = low anterior resection; APR = abdominoperineal resection; SD = standard deviation; reRT = reirradiation; IOERT = intraoperative electron radiation therapy; IQR = interquartile range; Gy = gray; RT = radiotherapy. *Other: (1) Dosage scheme: 6 × 2 Gy + 19 × 1.8 Gy due to acute bowel toxicity, (2) dosage scheme: 25 × 2 Gy + 10 Gy boost on pathological lymph nodes bilaterally. **Missing (*n* = 6)

### Surgery with IOERT

Surgical and IOERT characteristics are illustrated in Table 2. Median time between end of reirradiation and IOERT was 13 weeks (IQR 11–14). The median LRRC tumour EQD2Gy was 63 Gy (IQR 6–65 Gy, α/β = 10 Gy). Median cumulative tumour EQD2Gy was 113 Gy (IQR 94–115 Gy, α/β = 10 Gy). Post-operative complications are summarised in Supplementary Table S1. A 30-day (any Clavien-Dindo grade) post-operative complication rate of 78% was observed, with wound complications accounting for 15%, predominantly grade 1–2 (12.5%). Major post-operative complication (Clavien-Dindo 3b-5) rate was 20%.

**Table 2:** Surgical and intra-operative radiation therapy characteristics

		*n* = 40	%
ASA classification	I II III	1 34 5	3 85 13
Surgical procedure	LAR APR Total exenteration Resection n.o.s.	2 13 22 3	5 33 55 8
Blood loss (L)	Median (IQR)	2.5 (1.5–4.1)	
Time surgery (h)	Median (IQR)	6 (5–7)	
Additional resection performed	No Yes Bladder Pelvic side wall Prostate Vesicles Sacrum Ureter Vagina Uterus Ovaria	3 37 23 20 15 17 17 10 5 4 2	8 93 58 50 38 43 43 25 13 10 5
Site of IOERT boost*	Posterior Lateral left Infralevator Posterolateral right Lateral right	5 18 2 3 16	13 45 5 8 40
Diameter IOERT applicator (cm)	Median (IQR)	6 (5–6)	
IOERT dose at depth (Gy)	Median (IQR)	10 (10–10)	
IOERT dose at surface (Gy)	Median (IQR)	16 (16–16)	
EQD2Gy cumulative dose (total) *α/β = 10 Gy*	Median (IQR)	113 (94–115)	
EQD2Gy cumulative dose (current tumour) *α/β = 10 Gy*	Median (IQR)	63 (63–65)	
IOERT surface (cm^2^)	Median (IQR)	40 (28–40)	
Radicality	R0 R1	30 10	75 25

Due to rounding, not all percentages added up to 100% Abbreviations: LAR = low anterior resection; APR = abdominoperineal resection; resection n.o.s. = resection not otherwise specified; IOERT = intraoperative electron radiation therapy; IQR = interquartile range; L = liters; h = hours; Gy = gray

*Multiple locations possible. IOERT site location is subjectively classified due to the absence of standardized definitions

### Acute and late toxicity

All patients completed the planned neoadjuvant chemo-reirradiation. One hospitalisation related to reirradiation-induced toxicity (CTCAE ≥ grade 3) occurred; patient was admitted with grade 3 radiation enteritis, managed conservatively. All patients proceeded to surgery and IOERT.

**Fig. 1:**
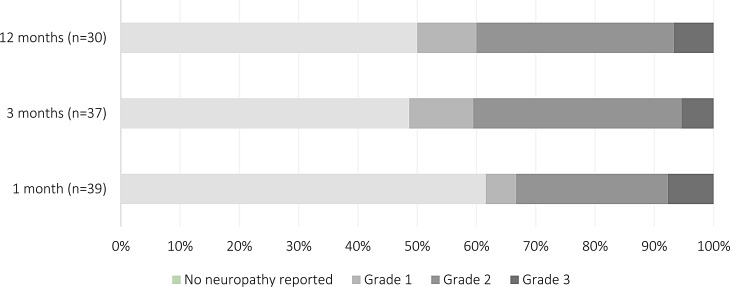
Total reported peripheral neuropathy over time, according to CTCAE grade

Worst-graded treatment-related acute and late toxicity events following multimodality treatment are illustrated in Table 3. A total of 37/40 patients reached 3-month follow-up, and 30/40 12-month follow-up. No grade 4 or 5 acute or late toxicities were observed. Acute grade 3 toxicities occurred in 14/37 (38%) patients. The main acute reirradiation-related toxicity was peripheral neuropathy, demonstrated in Fig. 1 and Supplementary Table S2. Grade 1–2 peripheral neuropathy occurred in 17/37 (46%) patients and grade 3 in 2/37 (5%) patients, of which four patients already reported neuropathy at baseline due to prior chemotherapy or surgery. Other commonly reported grade 3 acute toxicity included erectile dysfunction (5/37, 14%) and intra-abdominal or presacral abscess (4/37, 11%). Delayed wound healing was uncommon (2/37, 6%).

**Table 3:** Worst-grade recorded treatment-related acute and late toxicities, according to CTCAE grade (version 5.0)

		Grade 1	Grade 2	Grade 3
Acute toxicity (n = 37)*				
Gastro-intestinal disorders	Gastroparesis			2
	Nausea/weight loss		1	
Abdominal infections	Intra-abdominal abscess		1	2
	Presacral abscess		1	2
Reproductive system	Erectile dysfunction	2	2	5
Renal and urinary disorders	Urinary tract obstruction		5	
	Urinary retention		2	
	Urinary incontinence		1	
	Acute kidney injury		1	
	Bladder perforation		1	
Injury, procedural complications	Delayed wound healing		1	
	Wound dehiscence			1
Nervous system disorders	Peripheral neuropathy	4	13	2
Late toxicity (n = 30)**				
Gastro-intestinal disorders	Diarrhoea		1	
	Nausea	1	1	
Abdominal infections	Presacral abscess			1
Reproductive system	Erectile dysfunction	1	2	5
Renal and urinary disorder	Urinary tract obstruction		3	1
	Urinary incontinence	1	1	
	Acute kidney injury			2
	Urinary tract infection			1
	Urinary fistula			1
Injury, procedural complications	Delayed wound healing		1	
Nervous system disorder	Peripheral neuropathy	3	9	2

*n = 37/40. Three patients died within three months (*n* = 1 intraoperatively due to ventricular fibrillation, *n* = 1 postoperatively due to compartment syndrome, *n* = 1 at 2 months with unknown cause)

**n = 30/40. Five patients died within 12 months. Three patients did not yet reach 12-month follow-up. Two patients were lost to follow-up

Late grade 3 toxicities occurred in 13/30 (43%) patients. The main late reirradiation-related toxicity was also peripheral neuropathy. Grade 1–2 peripheral neuropathy occurred in 13/30 (43%) patients and grade 3 in 2/30 (7%) patients. Of the two late grade 3 peripheral neuropathies, one likely resulted from perioperative sacral plexus dissection (onset ≥ 1 month), while the other emerged at 12 months attributable to re-recurrence with ingrowth into lumbosacral plexus and sciatic nerves bilaterally. Overall, among patients with peripheral neuropathy (any grade) at 3 months, the majority 13/19 (68%) demonstrated improvement or stability of complaints at 12 months. Remaining patients were either lost to follow-up or had not yet reached the 12-month assessment. Longitudinal data on peripheral neuropathy are presented in the Supplementary Table S3. Other grade 3 late toxicities included erectile dysfunction (5/30, 17%) and renal and urinary disorders (5/30, 17%).

### Survival and local control

**Fig. 2:**
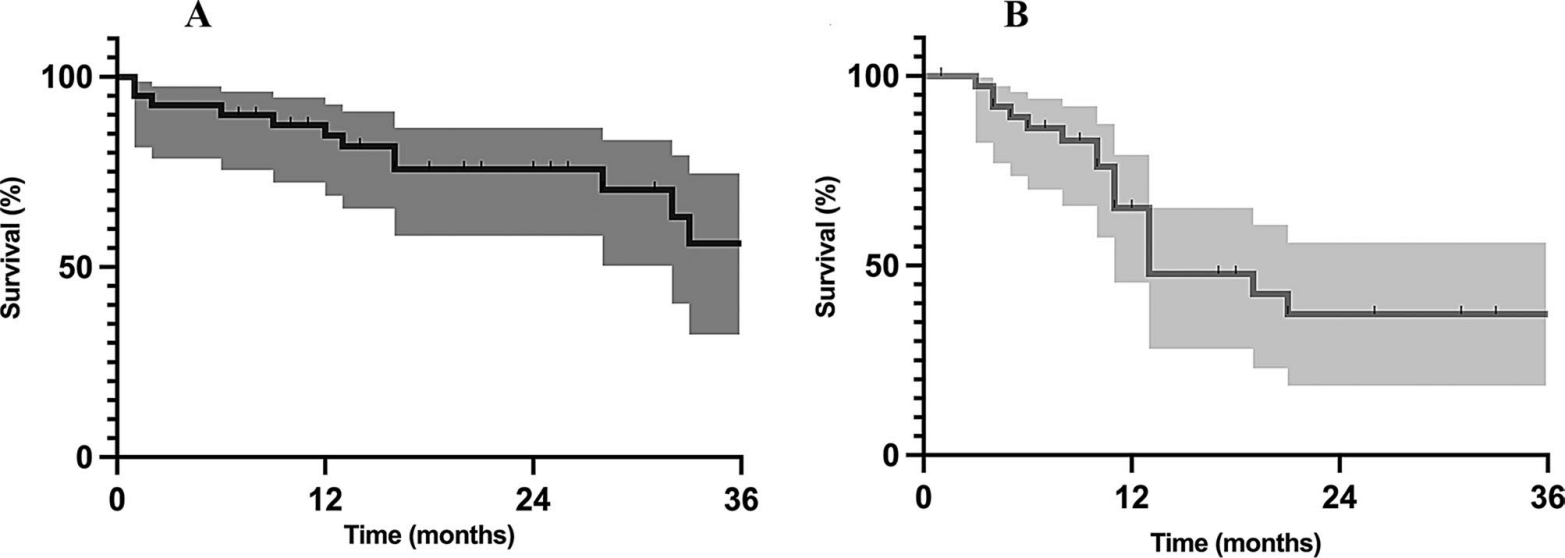
Kaplan-Meier estimates of (**A**) overall survival and (**B**) local re-recurrence-free survival with 95% confidence interval for LRRC patients undergoing chemo-reirradiation and surgery with IOERT

OS and LRFS are illustrated in Fig. 2. LRRC patients undergoing reirradiation and surgery with IOERT demonstrated a 1-, 2- and 3-year OS of 84.5%, 75.7% and 56.2%, respectively. Local control, defined as LRFS, was 65.1% and 37.2% at 1- and 2-years, respectively.

## Discussion

The current study evaluated the cumulative treatment-related toxicity and oncological outcomes of the multimodality approach of chemo-reirradiation and surgery with IOERT in LRRC patients. All patients received primary chemoradiation 25 × 2 Gy or 5 × 5 Gy and chemo-reirradiation 15 × 2 Gy with an IORT boost of 16 Gy, delivering a median cumulative tumour dose of 113 Gy (α/β = 10 Gy). Despite concerns regarding cumulative radiation dose and associated toxicities, no acute or late grade 4/5 toxicities were observed. Although grade 3 toxicity rates were marginally higher than prior series, it remained within clinically acceptable limits. Importantly, a low incidence of acute and late grade 3 peripheral neuropathy was observed. Favourable survival and disease control outcomes were observed, with 3-year OS of 56.2% and 2-year LRFS of 37.2%.

Prior studies have established that reirradiation is feasible and complements the likelihood of radical resection [13, 23]. A meta-analysis including 744 patients reported pooled rates of grade ≥ 3 acute and late complications of 11.7% and 25.5% [36]. Reirradiation with surgery demonstrated increased risk of grade ≥ 3 late complications (odds ratio: 6.4) [36]. Notably, hyperfractionated chemo-reirradiation has demonstrated low rates of acute toxicity and late complications [19]. As the biological rationale for hyperfractionation leverages the differential sensitivity of tumour and late-reacting normal tissues with lower α/β ratios, a potentially more favourable therapeutic ratio can be achieved. In reirradiation, this approach may be particularly beneficial for dose-escalation. Considering that hyperfractionation is rarely combined with IOERT, our centre historically has more experience with normofractionated reirradiation (with a lower dose 30 Gy versus 40.8 Gy [19]) which has demonstrated feasibility and low toxicity [13, 22].

Currently, literature on the toxicity of chemo-reirradiation combined with surgery and IOERT remains scarce. The present study observed acute and late treatment-related toxicities in 38% and 43%. Notably, acute and late grade 3 peripheral neuropathy was observed in 5% and 7%, within range of previously reported rates of 3% to 23% [26, 37]. A retrospective series by Haddock et al., reported grade 3 toxicities in 48%, with grade 3 peripheral neuropathy in 8% [38]. A larger series observed grade 3 toxicity or higher partially attributable to IOERT in 11%, with grade 3 peripheral neuropathy in 3% particularly at doses > 12.5 Gy [12]. Dijkstra et al. reported a post-operative grade 3 toxicity rate of 25.7% and acute and late neurological toxicity rates of 42.9% and 17.1% (any grade), respectively [20]. However, only 14 of 35 patients in this cohort received IOBT, complicating direct comparison. As the current study does not isolate chemo-reirradiation and IOERT toxicity separately, but rather reflects the clinically-relevant multimodality approach with extensive surgery, standardised registered toxicity rates may be marginally higher than prior series. The relatively low incidence of grade 3 peripheral neuropathy in this cohort suggests that, even within an intensified treatment regimen, this toxicity remains clinically manageable. Currently, dose tolerance of sacral nerves and cauda equina is not well defined and neural recovery between radiation courses may allow for higher cumulative doses than traditionally reported. As indirect guidance for peripheral nerve tolerance, Emami et al. reported normal tissue tolerance threshold of < 50 Gy (α/β = 2 Gy) for the spinal cord and < 60 Gy for the brachial plexus (α/β = 3 Gy) [39]. Specifically for sacral nerves, however, Abusaris et al. reported median cumulative doses up to 106 Gy (range 80–149 Gy, α/β = 3 Gy) without prohibitive toxicity [40]. In this study, median single-fraction IOERT boost dose (α/β = 3 Gy) was 26 Gy at depth and 60.8 Gy at surface, resulting in similar cumulative range of 106–140.8 Gy. Within this patient population, multimodality treatment inherently pushes boundaries of acceptable dose constraints, where a higher toxicity risk must be weighed against the risk of re-recurrence and associated morbidity. Future research on the integration of intraoperative surgical navigation [41–44] may provide further insights by enabling precise target volume delineation, facilitating the identification and avoidance of high-risk anatomical structures, ultimately mitigating toxicity risks.

Ultimately, the goal of multimodality radiation therapy is to improve OS and LRFS. The observed 1- and 3-year OS of 84.5% and 56.2%, respectively aligns with previous literature, though long-term survival in this study appears slightly favourable. Haddock et al. reported a 1- and 3-year OS of 74.9% and 28%, respectively [38]. A series by Vermaas et al. of 11 patients, observed a 1- and 3-year OS of 77% and 51%, respectively [24]. More recent pooled analyses by Holman et al. observed a 3-year OS of 52%, however, this included both irradiated and radiotherapy-naïve patients undergoing IOERT, complicating comparison [13]. Similarly, the observed 1- and 2-year LRFS of 65.1% and 37.2% are consistent with literature, such as Vermaas et al. (1- and 3-year LRFS of 66% and 27%) [24], and Haddock et al. (2-year LRFS of 39%) [38]. Variations in local control may be attributed to differences in patient selection, reirradiation dose, waiting period after preoperative chemo-reirradiation and the extent of surgical intervention.

The main strength of the current study is the selection of LRRC patients undergoing protocolised multimodality treatment with standardised prospective cumulative treatment-related toxicity registration. However, several limitations must be acknowledged. The absence of prospective toxicity data on chemo-reirradiation alone limits the ability to attribute toxicity to one modality only. Furthermore, a lack of control group complicates comparison. Nonetheless, this study provides insights into multimodality therapy, where the cumulative treatment-related toxicity is of primary clinical relevance. Despite implementation of a prospectively maintained database, a small risk of underreporting due to missing data remains. However, there is no reason to suspect that the missing data would significantly change current results. A longer follow-up period in a larger cohort would be necessary to analyse prognostic factors associated with toxicity and improved OS and LRFS. The introduction of the PelvEx II trial and shift in surgical approaches over time may also have altered the characteristics of patients with LRRC. Nonetheless, it is unlikely to obscure a clinically relevant or severe increase in toxicity following multimodality treatment. Forthcoming work will integrate data from image-guided surgical navigation of IOERT beam modelling to enhance the understanding of toxicity aetiology and in-field local failure evaluation [44]. While alternative reirradiation strategies, such as proton beam therapy and carbon ion therapy have also demonstrated feasibility [45, 46], their widespread clinical implementation remains constrained by limited data, accessibility, and cost-effectiveness. Given these challenges, further research on multimodality approach outlined in this paper is essential to optimising treatment strategies and outcomes.

## Conclusion

In previously irradiated LRRC patients, a multimodality curative approach combining chemo-reirradiation and surgery with IOERT demonstrated acceptable treatment-related toxicities and favourable oncological outcomes for this high-risk population. Further research with longer follow-up is warranted to identify risk factors associated with treatment-related toxicity.

## Supplementary Information

Below is the link to the electronic supplementary material.

Supplementary Material 1

Supplementary Material 2

Supplementary Material 3

## Data Availability

Data that support the findings of this study are available from the corresponding author upon reasonable request.

## Abbreviations

APR: Abdominoperineal resection
CT: Computed tomography
CTCAE: Common Terminology Criteria for Adverse Events (version 5.0)
CTV: Clinical target volume
EQD2Gy: Equivalent uniform dose in 2-Gy fraction
FDG-PET: Fluorodeoxyglucose-Positron Emission Tomography
GTV: Gross tumour volume
IOBT: Intraoperative brachytherapy
IOERT: Intraoperative electron radiotherapy
IQR: Interquartile range
LAR: Low anterior resection
LRFS: Local re-recurrence-free survival
LRRC: Locally recurrent rectal cancer
MDT: Multidisciplinary team
MRI: Magnetic resonance imaging
OAR: Organs at risk
OS: Overall survival
PTV: Planning target volume
R0: Radical resection margin
TE: Total exenteration
