# Supplementary material for: Neoadjuvant chemo-reirradiation followed by resection and intraoperative electron beam radiotherapy: outcomes of multimodality treatment for locally recurrent rectal cancer
Source: Radiat Oncol. 2025 Dec 23;21:21. doi: 10.1186/s13014-025-02782-w (PMC12853834; doi:10.1186/s13014-025-02782-w)
Supplement: Supplementary file 1 — Supplementary Material 1 [file 13014_2025_2782_MOESM1_ESM.docx]

**Table S1** Postoperative complications, according to Clavien-Dindo classification.

|  |  | ***n = 40*** | **%** |
| --- | --- | --- | --- |
| Admission (days) | Median (IQR) | 11 (8-14) |  |
| Any post-operative complication (30 days) | Any grade* | 31 | 78 |
| Uro-genital complications | Any grade  Grade 1-2  Grade 3a-5 | 6  5  1 | 15 |
| Cardiopulmonary complications | Any grade  Grade 1-2  Grade 3a-5 | 10  8  2 | 25 |
| Wound complications | Any grade  Grade 1-2  Grade 3a-5 | 6  5  1 | 15 |
| Gastro-intestinal complications | Any grade  Grade 1-2  Grade 3a-5 | 16  9  7 | 40 |
| Ileus/gastroparesis | Any grade  Grade 1-2  Grade 3a-5 | 12  10  2 | 30 |
| Infectious (including abscess) | Any grade  Grade 1-2  Grade 3a-5 | 10  6  4 | 25 |
| Other complications | Any grade  Grade 1-2  Grade 3a-5 | 13  8  5 | 33 |
| Highest Clavien-Dindo (30 days) | Grade 0-2  Grade 3a  Grade 3b-5 | 27  4  8 | 68  10  20 |
| Readmission (30 days) | No  Yes | 34  6 | 85  15 |
