# Supplementary material for: Neoadjuvant chemo-reirradiation followed by resection and intraoperative electron beam radiotherapy: outcomes of multimodality treatment for locally recurrent rectal cancer
Source: Radiat Oncol. 2025 Dec 23;21:21. doi: 10.1186/s13014-025-02782-w (PMC12853834; doi:10.1186/s13014-025-02782-w)
Supplement: Supplementary file 2 — Supplementary Material 2 [file 13014_2025_2782_MOESM2_ESM.docx]

**Table S2:** Reported peripheral neuropathy over time, according to CTCAE grading (version 5.0).

|  |  | *n* | % |
| --- | --- | --- | --- |
| Physician-reported neuropathy | 30 days (*n =* 39)  Grade 0  Grade 1-2  Grade 3 | 24  12  3 | 62  31  7 |
|  | 3 months (*n =* 37)  Grade 0  Grade 1-2  Grade 3 | 18  17  2 | 49  46  5 |
|  | 12 months (*n =* 30)  Grade 0  Grade 1-2  Grade 3 | 15  13  2 | 50  43  7 |

*Due to rounding, not all percentages added up to 100%.*
