# Supplementary material for: Neoadjuvant chemo-reirradiation followed by resection and intraoperative electron beam radiotherapy: outcomes of multimodality treatment for locally recurrent rectal cancer
Source: Radiat Oncol. 2025 Dec 23;21:21. doi: 10.1186/s13014-025-02782-w (PMC12853834; doi:10.1186/s13014-025-02782-w)
Supplement: Supplementary file 3 — Supplementary Material 3 [file 13014_2025_2782_MOESM3_ESM.docx]

**Table S3:** Longitudinal data of peripheral neuropathy over time, according to CTCAE grading.

| **ID** | **Baseline (n=40)** | **30 days (n=39)** | **3 months (n=37)** | **12 months (n=30)** |
| --- | --- | --- | --- | --- |
| 1 | Grade 0 | Grade 0 | Grade 0 | Grade 3 |
| 2 | Grade 0 | Grade 0 | Grade 0 | Grade 0 |
| 3 | Grade 0 | Grade 0 | Grade 0 | Grade 0 |
| 4 | Grade 2 | Grade 3 | Grade 3 | Grade 2 |
| 5 | Grade 0 | Grade 0 | Grade 2 | Grade 1 |
| 6 | Grade 0 | Grade 0 | Grade 1 | Grade 0 |
| 7 | Grade 0 | Grade 0 | Grade 0 | Grade 0 |
| 8 | Grade 0 | Grade 0 | Not reached due to death | |
| 9 | Grade 0 | Grade 0 | Grade 0 | Grade 1 |
| 10 | Grade 0 | Grade 0 | Grade 0 | Grade 2 |
| 11 | Grade 0 | Grade 0 | Grade 0 | Grade 0 |
| 12 | Grade 0 | Grade 2 | Grade 2 | Lost to follow-up |
| 13 | Grade 0 | Grade 0 | Grade 1 | Grade 0 |
| 14 | Grade 0 | Grade 0 | Not reached due to death | |
| 15 | Grade 0 | Grade 2 | Grade 2 | Not reached due to death |
| 16 | Grade 0 | Grade 0 | Grade 1 | Grade 0 |
| 17 | Grade 0 | Grade 0 | Grade 0 | Grade 0 |
| 18 | Grade 0 | Grade 0 | Grade 0 | Grade 0 |
| 19 | Grade 0 | Grade 0 | Grade 0 | Grade 2 |
| 20 | Grade 0 | Grade 0 | Grade 0 | Grade 2 |
| 21 | Grade 2 | Grade 3 | Grade 2 | Grade 2 |
| 22 | Grade 0 | Grade 0 | Grade 0 | Grade 0 |
| 23 | Grade 0 | Grade 3 | Grade 3 | Grade 3 |
| 24 | Grade 0 | Grade 0 | Grade 0 | Grade 0 |
| 25 | Grade 0 | Grade 2 | Grade 2 | Grade 2 |
| 26 | Grade 0 | Not reached due to death | | |
| 27 | Grade 0 | Grade 1 | Grade 2 | Grade 2 |
| 28 | Grade 0 | Grade 0 | Grade 0 | Grade 0 |
| 29 | Grade 2 | Grade 2 | Grade 2 | Grade 1 |
| 30 | Grade 0 | Grade 1 | Grade 1 | Not reached due to death |
| 31 | Grade 0 | Grade 2 | Grade 2 | Grade 2 |
| 32 | Grade 2 | Grade 2 | Grade 2 | Grade 2 |
| 33 | Grade 0 | Grade 0 | Grade 0 | Grade 0 |
| 34 | Grade 0 | Grade 2 | Grade 2 | Lost to follow-up |
| 35 | Grade 0 | Grade 0 | Grade 0 | Grade 0 |
| 36 | Grade 0 | Grade 2 | Grade 2 | Grade 2 |
| 37 | Grade 0 | Grade 0 | Grade 0 | Grade 0 |
| 38 | Grade 0 | Grade 2 | Grade 2 | Not yet reached |
| 39 | Grade 1 | Grade 0 | Grade 0 | Not yet reached |
| 40 | Grade 0 | Grade 2 | Grade 2 | Not yet reached |
